# Supplementary material for: Reconciling Mining with the Conservation of Cave Biodiversity: A Quantitative Baseline to Help Establish Conservation Priorities
Source: PLoS One. 2016 Dec 20;11(12):e0168348. doi: 10.1371/journal.pone.0168348 (PMC5173368; doi:10.1371/journal.pone.0168348)
Supplement: S1 Dataset — (ZIP) [file pone.0168348.s002.zip › Taxa/Serra Norte/SN_2013/N4E/Lista N4E-67.pdf]

CAVIDADE N4E-0067

Classe	Ordem	Fam/Outros	Gên/Outros	Espécie	S-U	U-S	Zon.
Annelida	Oligochaeta			jovem	1	1	E/P
Arachnida	Acari	Cunaxidae		sp.1	X		P
Arachnida	Acari	Gamasida		sp.1		X	E
Arachnida	Acari	Laelapidae		sp.1	X		P
Arachnida	Acari	Mesostigmata		jovem	X		E/P
Arachnida	Acari	Podocinidae	*Podocinum*	sp.1	X		P
Arachnida	Acari	Trombiculidae		sp.39		X	E
Arachnida	Acari	Trombiculidae		sp.41	X		P
Arachnida	Acari	Trombidiidae		sp.65	X		P
Arachnida	Amblypygi	Charinidae	*Charinus*	sp.1	1		E
Arachnida	Amblypygi	Phrynidae	*Heterophrynus*	*longicornis*		2	E
Arachnida	Araneae	Araneidae		jovem		X	E
Arachnida	Araneae	Barychelidae		jovem		1	
Arachnida	Araneae	Corinnidae		jovem		2	E/P
Arachnida	Araneae	Ctenidae	*Ctenus*	sp.1	1	1	E
Arachnida	Araneae	Pholcidae	*Mesabolivar*	sp.1	X	X	E/P
Arachnida	Araneae	Salticidae		jovem	X		E
Arachnida	Araneae	Scytodidae	*Scytodes*	*eleonorae*	2	8	E
Arachnida	Araneae	Theraphosidae	aff. *Holothele*	sp.1	2		
Arachnida	Araneae	Theridiidae	*Episinus*	sp.1	X		E
Arachnida	Araneae	Theridiidae	*Theridion*	sp.1		X	E
Arachnida	Araneae	Theridiosomatidae	*Plato*	sp.1	X	X	E/P
Arachnida	Pseudoscorpiones	Bochicidae		sp.1	X		P
Arachnida	Pseudoscorpiones	Cheliferoidea		sp.1		X	P
Arachnida	Pseudoscorpiones	Chthoniidae	*Pseudochthonius*	sp.1	X	X	E/P
Arachnida	Pseudoscorpiones	Olpiidae		sp.1		X	E
Diplopoda	Polydesmida	Pyrgodesmidae		sp.1		X	P
Entognatha	Collembola	Paronellidae		sp.4	X		P
Entognatha	Collembola	Sminthuroidea		sp.2	X	X	E/P
Insecta	Blattodea	Blattellidae		jovem	1	4	E
Insecta	Coleoptera	Staphylinidae		sp.1	X	X	P
Insecta	Diptera	Chironomidae		sp.	X		E
Insecta	Diptera	Dolichopodidae		sp.		X	P
Insecta	Diptera	Drosophilidae	*Drosophila*	*eleonorae*	X		E
Insecta	Diptera	Limoniidae		sp.	X		E
Insecta	Diptera	Psychodidae	Phlebotominae	sp.	X	X	E/P
Insecta	Diptera	Sciaridae		sp.	X		P
Insecta	Diptera	Sepsidae		sp.	X		P
Insecta	Diptera	Streblidae	*Trichobius*	sp.	X	X	E/P
Insecta	Diptera	Tipulidae	Tipulinae	sp.	X		E
Insecta	Homoptera	Cixiidae		jovem		X	P
Insecta	Hymenoptera	Formicidae	*Camponotus*	sp.2		X	P
Insecta	Hymenoptera	Formicidae	*Cyphomyrmex*	sp.1		X	P
Insecta	Hymenoptera	Formicidae	*Eurhopalothrix*	sp.2		X	P
Insecta	Hymenoptera	Formicidae	*Pachycondyla*	sp.2	X	X	E/P
Insecta	Hymenoptera	Formicidae	*Solenopsis*	sp.1		X	E
Insecta	Hymenoptera	Formicidae	*Trachymyrmex*	sp.1		X	P
Insecta	Isoptera	Termitidae	*Nasutitermes*	sp.	X	X	E
Insecta	Lepidoptera	Tineoidea		sp.2	X	X	E

Insecta	Lepidoptera	Tineoidea		sp.3	X		E
Insecta	Mantodea	Mantidae		sp.1		2	E
Insecta	Orthoptera	Gryllidae	Nemobiinae	sp.		2	
Insecta	Orthoptera	Phalangopsidae	*Phalangopsis*	sp.	50	85	E/P
Insecta	Psocoptera	Epipsocidae	*Epipsocus*	sp.2	X		E
Insecta	Thysanura	Nicoletiidae		sp.1	X	X	E/P
Gastropoda	Pulmonata	Bulimulidae	*Naesiotus*	sp.	X		E
Malacostraca	Isopoda	Armadillidae		sp.1	X	X	P
Malacostraca	Isopoda	Philosciidae		sp.1		X	E
Symphyla		Scutigerellidae		sp.	X		E
Amphibia	Anura	Strabomantidae	*Pristimantis*	cf. *fenestratus*	8	1	E/P
Mammalia	Chiroptera			sp.	4	6	E/P
Reptilia	Squamata	Gekkonidae	*Thecadactylus*	sp.	1		E
